# Organic-Inorganic Hybrid Silica Material Derived from a Monosilylated Grubbs-Hoveyda Ruthenium Carbene as a Recyclable Metathesis Catalyst

**DOI:** 10.3390/molecules15085756

**Published:** 2010-08-23

**Authors:** Guadalupe Borja, Roser Pleixats, Ramón Alibés, Xavier Cattoën, Michel Wong Chi Man

**Affiliations:** 1 Chemistry Department, Universitat Autònoma de Barcelona, Cerdanyola del Vallès, 08193-Barcelona, Spain; 2 Institut Charles Gerhardt Montpellier (UMR 5253 CNRS-UM2-ENSCM-UM1), Architectures Moléculaires et Matériaux Nanostructurés, Ecole Nationale Supérieure de Chimie de Montpellier, 8 rue de l’école normale, 34296 Montpellier cédex 5, France

**Keywords:** catalyst immobilization, ring-closing metathesis, organic-inorganic hybrid material, ruthenium alkylidene, sol-gel process

## Abstract

The synthesis of a monosilylated Grubbs-Hoveyda ruthenium alkylidene complex is described, as well as the preparation and characterization of the corresponding material by sol-gel cogelification with tetraethoxysilane (TEOS) and the assay of this recyclable supported catalyst in ring-closing diene and enyne metathesis reactions under thermal and microwave conditions.

## 1. Introduction

**Figure 1:**
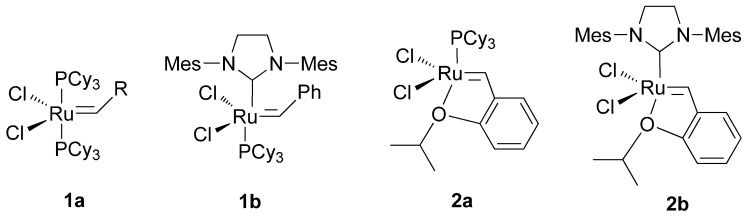
Ruthenium carbene metathesis catalysts.

Olefin metathesis is a very powerful, mild, efficient, versatile and selective method for the cleavage and the formation of C-C double bonds, which has been widely used by organic chemists for the preparation of a great variety of compounds and polymers [1,2,3,4,5,6,7,8,9,10,11,12]. Ring-closing enyne metathesis has also been explored in recent years as an atom economical process to provide 1-vinylcycloalkenes from acyclic enynes [13,14,15,16,17,18,19]. The enormous success of metathesis procedures in the academic field during the last decade is due to the development of several well-defined metal alkylidenes. The second-generation Grubbs ruthenium catalysts [20] **1b** (Figure 1) and especially the Grubbs-Hoveyda catalysts [21,22] **2a,b** (Figure 1) show enhanced reactivity, stability and recovery profiles compared to the first generation Grubbs catalyst **1a** (Figure 1), the chelating ligand playing a crucial role in such improvements. 

On the other hand, the increased robustness and stability of the Hoveyda-Grubbs carbenes facilitates the preparation of recyclable metathesis catalysts [23,24,25,26]. One of the most commonly used recycling strategies consists of the immobilization of the alkylidene complex on an insoluble polymeric support. An easy separation of the product and recovery of the catalyst can then be achieved by simple filtration at the end of the reaction, avoiding time-consuming chromatography. Anchoring of ruthenium complexes of type **1** and **2** to the polymeric support can be performed via alkylidene exchange (*boomerang*-type catalysts). The efficiency of *boomerang* supported catalysts increases notably when Hoveyda-type ligands are involved (release-return mechanism) [27]. Insoluble organic polymers have mainly been used as supports, and in the context of catalyst recycling, it is worth to mention that hybrid organic-inorganic silica materials show chemical, mechanical and thermal stability superior to that of organic polymers and, most often, higher surface areas. To date only a few examples of silica-bound alkylidene complexes have been described [23,24,25,26,28,29,30] and these silica-bound metathesis catalysts always refer to anchoring the metal containing moiety to pre-formed porous or non-porous silicas. Despite the fact that sol-gel hydrolytic condensation [31] of suitable organo-alkoxysilanes is a convenient method to prepare solid hybrid materials with targeted properties [32,33], no precedents are available in the literature about the formation of a hybrid silica material via such a process starting from a silylated Grubbs ruthenium-alkylidene complex. We have previously reported preparation of several efficient and recyclable Grubbs-Hoveyda type heterogenized metathesis catalysts through sol-gel procedures on suitably modified Hoveyda ligands [34,35,36]. However, in these cases, the sol-gel process was first performed on the silylated monomeric ligands and the metal was subsequently introduced in the synthesized material. Very recently, some of us have also described DFT mechanistic studies on the catalytic activity and catalyst recovery of these supported catalysts [37]. 

We present herein the synthesis of a monosilylated Grubbs-Hoveyda ruthenium alkylidene complex, the preparation and characterization of the corresponding material by sol-gel cogelification with tetraethoxysilane (TEOS) and the assay of this supported catalyst in ring-closing diene and enyne metathesis reactions under thermal and microwave conditions. 

## 2. Results and Discussion

### 2.1. Synthesis of the monosilylated monomer and preparation of hybrid silica material M1

**Scheme 1:**
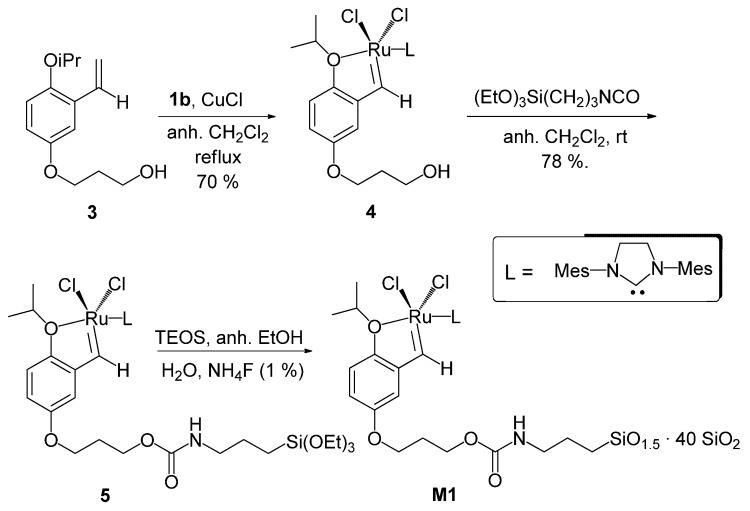
Preparation of monosilylated ruthenium complex **5** and hybrid material **M1**.

The monosilylated monomer **5** required for the synthesis of the hybrid material **M1** was prepared as summarized in Scheme 1. Treatment of the known alcohol **3** [35] with the second generation Grubbs catalyst **1b** in refluxing anhydrous dichloromethane in the presence of CuCl as phosphine scavenger [22] gave the ruthenium complex **4. **This wasobtained as a green solid in 70% isolated yield after chromatography of the crude mixture through silica gel and was fully characterized by IR, ^1^H-NMR, ^13^C-NMR and HR-MS. Subsequent reaction of the alcohol **4** with commercial 3-(isocyanatopropyl)-triethoxysilane in anhydrous dichloromethane at room temperature afforded the desired silylated carbamate **5** in 78% isolated yield, also as a green solid. Characterization of **5** was accomplished by ^1^H-NMR, ^13^C-NMR, ESI-MS and HR-MS-FAB. Bidimensional NMR experiments (^1^H-^1^H COSY, ^1^H-^1^H NOESY, ^1^H-^13^C HSQC, ^1^H-^13^C HMBC) allowed the complete assignment of the signals in the ^1^H NMR spectra for both **4** and **5**. 

**Figure 2:**
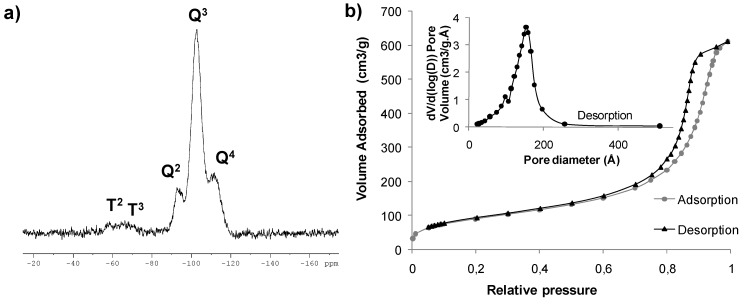
**(a)** Solid state CP-MAS ^29^Si NMR spectrum of **M1**. **(b)** N_2_ sorption isotherm of **M1** and plot of the pore size distribution.

Co-gelification of **5** with TEOS (molar ratio 1:40) was performed in anhydrous ethanol at room temperature under nucleophilic conditions (one equivalent of water per ethoxy group, 1% molar of ammonium fluoride as catalyst). The solution gelified overnight and it was allowed to age for five days. Then, it was washed successively with ethanol and dichloromethane, and the powder was dried overnight under vacuum at 60 ºC to afford **M1** as a green solid (Scheme 1). This material was studied by several techniques (^29^Si CP-MAS NMR, N_2_ adsorption-desorption analysis, elemental analysis, ICP). The ^29^Si CP-MAS NMR of **M1** confirmed the covalent bonding of the organic moiety to the matrix by the presence of T^2^ and T^3^ signals at -57.0 and -64.1 ppm respectively, in addition to the characteristic Q^2^, Q^3^ and Q^4^ signals due to the condensed TEOS at -92.9, -102.6 and -111.7 ppm (Figure 2a). The N_2_ adsorption-desorption isotherm of **M1** is representative of a material with large mesopores (pore size distribution centered around 120–175 Å), with a total pore volume of 0.89–0.93 cm^3^/g and a BET surface area of 332 m^2^/g (Figure 2b). The ruthenium content was determined by inductively coupled plasma (ICP) analysis (0.86% w/w, 0.085 mmol Ru/g of material). The results of elemental analysis revealed a N/Si ratio of 1/20 and a Ru/N ratio of about 1/6. As the theoretical values should be 1/14 and 1/3 respectively considering a complete condensation of the monomer, it is likely that incomplete condensation and partial loss of the metal has occurred during the formation of the material by the sol-gel process**. **

### 2.2. Catalytic activity and recyclability of the hybrid material **M1** in diene and enyne ring-closing metathesis reactions

**Scheme 2:**
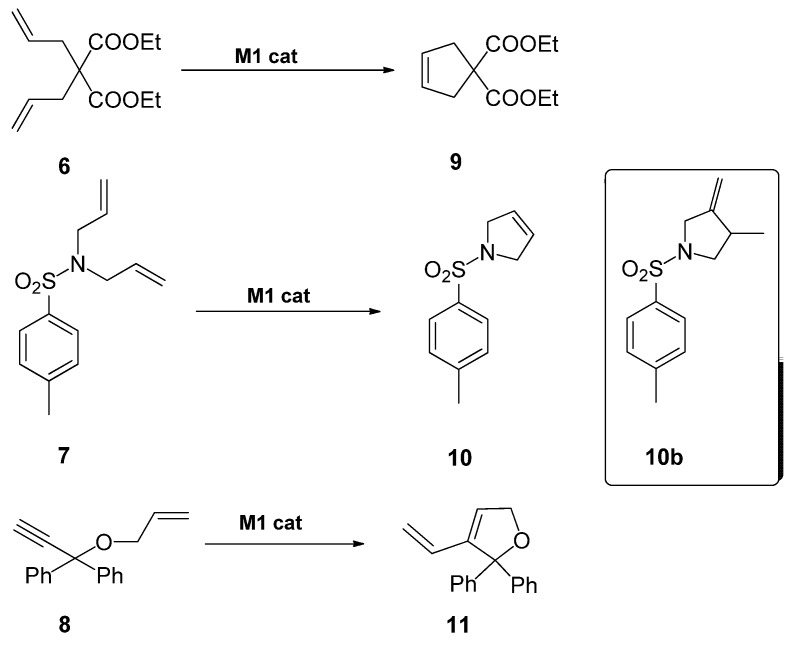
Diene and enyne metathesis reactions tested with hybrid material **M1**.

The catalytic activity of the material **M1** was tested for the ring-closing metathesis reactions on diene and enyne substrates **6**, **7** and **8** (Scheme 2). The results are summarized in Table 1. 

**Table 1:** Results for the diene and enyne RCM reactions with hybrid material **M1**.**^ a^**

Entry	Substrate	Solvent	Heating	T (ºC)	t (h)	Cycle	Conversion % ^b^
1	**6**	CH_2_Cl_2_	--	rt	20	1	100
2	**6**	CH_2_Cl_2_	Conventional	reflux	24	2	100
3	**6**	CH_2_Cl_2_	MW^c^	60	0.33	1	100
4	**6**	CH_2_Cl_2_	MW^c^	60	0.33	2	97
5	**6**	CH_2_Cl_2_	MW^c^	60	0.33	3	94
6	**6**	CH_2_Cl_2_	MW^c^	60	0.33	4	79
7	**6**	CH_2_Cl_2_	MW^c^	60	2.33	5	77
8	**7**	CH_2_Cl_2_	--	rt	24	1	94
9	**7**	CH_2_Cl_2_	conventional	reflux	24	1	100^d^
10	**7**	toluene	--	rt	24	1	88
11	**7**	CH_2_Cl_2_	MW^c^	50	0.67	1	84^e^
12	**7**	CH_2_Cl_2_	MW^c^	45	4	2	65^f^
13	**8**	CH_2_Cl_2_	conventional	reflux	22	1	100
14	**8**	CH_2_Cl_2_	MW^c^	60	3	1	100
15	**8**	CH_2_Cl_2_	MW^c^	60	15	2	64

^a^ General conditions: anhydrous and degassed solvent, inert atmosphere, 3.5 mol% of Ru, [substrate] = 0.05 M. ^b^ Conversion determined by NMR, only the desired product being formed unless otherwise stated. ^c^ Microwave irradiation with *PowerMax* option enabled. ^d ^82% of **10 **and 18% of the cycloisomerization product **10b.** ^e^ 77% of **10 **and 7% of the cycloisomerization product **10b. **^f^ 63% of **10 **and 2% of the cycloisomerization product **10b.**

The intramolecular reaction on diethyl 2,2-diallylmalonate (**6**), performed in anhydrous and degassed dichloromethane at room temperature, gave product **9** with a 100% conversion after 20 h, with no other compound being detectable by NMR. The desired final product **9** was isolated in pure form after simple filtration and solvent evaporation (entry 1). The recovered catalyst was reused in a second cycle, but refluxing conditions were required to produce 100% conversion after 24 h (entry 2). Alternatively, microwave irradiation was used as a means of shortening the reaction times [38,39], which can be beneficial in order to avoid catalyst decomposition by prolonged heating. Thus, when the process was performed at 60 ºC under microwave irradiation, the reaction was complete in 20 min and the catalyst was reused up to 5 runs (entries 3-7). The activity was maintained for the first three cycles. The conversion decreased in the fourth cycle to 79% for the same reaction time (20 min) and higher reaction time (140 min) was required to achieve a similar conversion (77%) in the fifth cycle. Following these studies on the RCM of **6**, four different set of conditions were tested for the similar reaction on *N,N*-diallyl-*p*-toluenesulfonamide (**7**) to afford product **10** (entries 8-12). By performing the reaction in dichloromethane at room temperature a 94% conversion was achieved after 24 h (entry 8). Complete conversion was attained under refluxing dichloromethane, but thermal heating results in the formation of a secondary product **10b **derived from a cycloisomerization process (entry 9). A change of solvent from dichloromethane to toluene led to 88% conversion of **7** after 24 h at room temperature (entry 10). Although secondary product **10b** was avoided under these conditions, the reaction rate was not higher than in dichloromethane at the same temperature. Interestingly by adopting microwave irradiation at 50 ºC in dichloromethane in similar reaction conditions (entry 11), a 87% conversion was observed after 45 min, although the mixture still contained minor amounts of the secondary product **10b** (77% of **10** and 7% of **10b**). The recycling of the catalyst **M1** was studied under microwave irradiation at 45ºC, affording a conversion of 65% after 4 h. The mixture contained also a minor amount of **10b** (2%) (entry 12). Thus, it seems that prolonged heating or irradiation at higher temperatures must be avoided for the RCM reaction with this substrate **7** and catalyst **M1**. Otherwise, a competitive cycloisomerization reaction of **7** occurs. Cycloisomerization of a diene in competition to the expected ring-closing metathesis reaction has been previously reported [40], this side reaction being mediated by a decomposition product of the initial metathesis catalyst. It is not unlikely that ruthenium hydride species are involved in such transformations. Finally, an enyne RCM reaction was assayed with 1-allyloxy-1,1-diphenyl-2-propyne (**8**) to give the diene **11 **(entries 13-15). Under refluxing dichloromethane a complete conversion was observed after 22 h (entry 13). The reaction time required for 100% conversion was reduced considerably (3 h) by adopting microwave irradiation conditions at 60 ºC (entry 14). The recovered material **M1** was reused under analogous conditions to afford 64% conversion after 15 h (entry 15).

After the reaction, the ruthenium contents in the crude products **9** and **10** (Table 1, entries 4 and 8) corresponded to 353 ppm and 175 ppm, respectively, as determined by ICP analysis. Thus the loss of ruthenium in **M1** during a reaction cycle was 1.9% and 0.8%, respectively. 

Comparison of the results presented here with those previously described by us [35], where the sol-gel process was first performed on the silylated monomeric Hoveyda ligand and the metal was subsequently introduced in the synthesized material, show that the catalytic activity and recyclability was superior in our previous work. Diffusion problems could be on the origin of the lower activity of the herein described **M1**. Presumably the ruthenium complex is mainly located in the present case inside the material, being less accessible to the reactants. The requirement of more drastic conditions and the presence of silanol groups on the material could explain the formation of decomposition species leading to side reactions or catalyst deactivation [41]. Nevertheless, it is worth to mention that olefin metathesis with Hoveyda-Grubbs catalysts has been successfully performed in aqueous media, suggesting the high stability of this type of complexes [42]. 

## 3. Experimental

### 3.1. General

^1^H- and ^13^C-NMR spectra were recorded on a Bruker DPX250 or on a Bruker AVANCE-III 400 instrument and the *J* values are given in Hz. The CP-MAS ^29^Si solid state NMR spectra were recorded on a Bruker AV-400-WB at the Servei de Ressonància Magnètica Nuclear of the Universitat Autònoma de Barcelona. MS (ESI) and HR-MS (ESI) analyses were recorded at the Servei d’Anàlisi Químic of the Universitat Autònoma de Barcelona on a Hewlett-Packard 5989A instrument. HR-MS (FAB) analysis was recorded at the Unidad de Espectrometría de Masas of the Universidad de Santiago de Compostela using 3-nitrobenzyl alcohol as matrix. IR data (KBr) were obtained with a Thermo Nicolet IR200 spectrophotometer. Elemental analyses have been performed at the Serveis Científicotècnics of the Universitat de Barcelona, using Inductively Coupled Plasma (ICP) for Ru and Si. Surface areas were calculated using the Brunauer-Emmett-Teller (BET) method based on N_2_ adsorption-desorption studies performed on a Micromeritics ASAP2020 analyzer at the Institut Charles Gerhardt Montpellier, after degassing the material at 55 ºC for 30 h. Microwave reactions were conducted on a CEM Discover^®^ Microwave synthesizer. The machine consists of a continuous focused microwave-power delivery system with operator-selectable power output from 0 to 300 W. Reactions were performed in glass vessels (capacity 10 mL) sealed with a septum. Temperature measurements were conducted using an infrared temperature sensor mounted under the reaction vessel. All experiments were performed using a stirring option whereby the contents of the vessel were stirred by means of a rotating magnetic plate located below the floor of the microwave cavity and a Teflon-coated magnetic stir bar in the vessel. All experiments were carried out with simultaneous cooling by passing compressed nitrogen through the microwave cavity while heating (*PowerMAX* option enabled). The alcohol **3** was prepared as previously described [34].

### 3.2. Preparation of the Grubbs-Hoveyda ruthenium alkylidenic complex **4**

Complex **1b** (1.852 g, 2.18 mmol) and CuCl (216 mg, 2.18 mmol) were weighed into a Schlenk flask under an inert atmosphere and dissolved in anhydrous CH_2_Cl_2 _(42.5 mL). A solution of **3** (500 mg, 2.12 mmol) in anhydrous CH_2_Cl_2 _(45 mL) was added. The mixture was refluxed for 2 h under argon atmosphere (^1^H-NMR monitoring), as initial deep red colour turned green. From this point forth, all manipulations were carried out in air with reagent-grade solvents. The reaction mixture was concentrated under vacuum to give a dark green solid residue, which was dissolved in a minimal volume of 1:1 pentane/CH_2_Cl_2_. The solution was passed through a pipette containing a plug of cotton and purified by column chromatography through silica gel. Elution with CH_2_Cl_2_ to CH_2_Cl_2_/MeOH (98:2), removal of solvent and drying afforded **4** as a bright green solid (1.04 g, 70%). ^1^H-NMR (CDCl_3_, 250 MHz) δ (ppm): 16.45 (s, 1H, Ru=CH), 7.10 (m, 1H, aromatic CH), 7.07 (s, 4H, mesityl aromatic CH), 6.68 (d, 1H, aromatic CH, *J *= 9.3 Hz), 6.47 (d, 1H, aromatic CH, *J* = 3.0 Hz), 4.81 (sept, 1H, -CH(CH_3_)_2_, *J *= 6.0 Hz), 4.18 (m, 4H, -N-CH_2_-CH_2_-N-), 4.03 (t, 2H, -O-CH_2_-CH_2_-CH_2_-OH, *J* = 5.8 Hz), 3.85 (q, 2H, -O-CH_2_-CH_2_-CH_2_-OH, *J* = 5.5 Hz), 2.47 (s, 12H, mesityl CH_3_), 2.40 (s, 6H, mesityl CH_3_), 2.01 (quint, 2H, -O-CH_2_-CH_2_-CH_2_-OH, *J* = 5.9 Hz), 1.70 (t, 1H, -OH, *J* = 5.3 Hz), 1.24 (d, 6H, -CH(CH_3_)_2_, *J* = 5.8 Hz). ^13^C-NMR (CDCl_3_, 100.6 MHz) δ (ppm): 296.3 (Ru=CH), 211.3 (Ru-NHC), 154.2 (aromatic C), 146.7 (aromatic C), 145.6 (aromatic C), 138.9 (mesityl C), 129.4 (mesityl CH), 115.8 (aromatic CH), 113.3 (aromatic CH), 108.0 (aromatic CH), 75.0 (-CH(CH_3_)_2_), 66.7 (-O-CH_2_-CH_2_-CH_2_-OH), 60.5 (-O-CH_2_-CH_2_-CH_2_-OH), 51.6 (-N-CH_2_-CH_2_-N-), 32.1 (-O-CH_2_-CH_2_-CH_2_-OH), 21.2 (mesityl CH_3_+ -CH(CH_3_)_2_). IR ν (cm^-1^) (KBr): 3448, 2923, 1688, 1488, 1258, 1214, 855. m.p (ºC): 199 (dec.). HR-MS (ESI) Calcd. for [C_34_H_44_Cl_2_N_2_O_3_RuNa]^+^: 723.1652; Found: 723.1668.

### 3.3. Preparation of the monosilylated Grubbs-Hoveyda ruthenium alkylidenic complex **5**

Freshly distilled 3-isocyanatopropyltriethoxysilane (54 µL, 0.99 g/mL, 0.216 mmol) was added under argon to **4 **(150 mg, 0.214 mmol) in anhydrous dichloromethane (0.5 mL). The mixture was stirred under argon at room temperature for 4 days. The solvent was removed under vacuum. The residue was washed with anhydrous pentane to afford **5** (231 mg, 64%) as a green solid. ^1^H-NMR (CDCl_3_, 400 MHz) δ (ppm): 16.44 (s, 1H, Ru=CH), 7.06 (m, 5H, mesityl CH + aromatic CH), 6.67 (d, 1H, aromatic CH, *J* = 8.0 Hz), 6.45 (d, 1H, aromatic CH, *J *= 8.0 Hz), 4.80 (septet, 1H, -CH(CH_3_)_2_, *J *= 6.0 Hz), 4.17 (m, 4H, -N-CH_2_-CH_2_-N-), 4.03-3.92 (m, 4H, -O-CH_2_-CH_2_-CH_2_-O-), 3.81 (q, 6H, -O-CH_2_-CH_3, _*J* = 7.6 Hz), 3.17 (m, 2H, -NH-CH_2_-CH_2_-CH_2_-Si(OEt)_3_), 2.46 (s, 12H, mesityl CH_3_), 2.39 (s, 6H, mesityl CH_3_), 2.06-2.00 (m, 2H, -O-CH_2_-CH_2_-CH_2_-O-), 1.63 (m, 2H, -NH-CH_2_-CH_2_-CH_2_-Si(OEt)_3_), 1.22 (m, 15H, -CH(CH_3_)_2 _+ -O-CH_2_-CH_3_), 0.63 (m, 2H, -NH-CH_2_-CH_2_-CH_2_-Si(OEt)_3_). ^13^C-NMR (CDCl_3_, 100.6 MHz) δ (ppm): 296.3 (Ru=CH), 211.4 (Ru-NHC), 154.3 (aromatic C), 146.8 (aromatic C), 145.6 (aromatic C), 138.9 (mesityl C), 129.5 (mesityl CH), 115.8 (aromatic CH), 113.2 (aromatic CH), 108.1 (aromatic CH), 75.0 (-CH(CH_3_)_2_), 66.7 (-O-CH_2_-CH_2_-CH_2_-O- or -O-CH_2_-CH_2_-CH_2_-O-), 60.5 (-O-CH_2_-CH_2_-CH_2_-O- or -O-CH_2_-CH_2_-CH_2_-O ), 58.6 (-O-CH_2_-CH_3_), 51.6 (-N-CH_2_-CH_2_-N-), 43.1 (-NH-CH_2_-CH_2_-CH_2_-Si(OEt)_3_), 32.2 (-O-CH_2_-CH_2_-CH_2_-O-), 22.4 (-NH-CH_2_-CH_2_-CH_2_-Si(OEt)_3_), 21.1 (mesityl CH_3_+ -CH(CH_3_)_2_), 18.4 (-O-CH_2_-CH_3_). 7.7 (-NH-CH_2_-CH_2_-CH_2_-Si(OEt)_3_). ESI-MS m/z (rel. int. %): [M–Cl]^+^ 912.3 (100), [M–2Cl]^+ ^876.4 (33.5). HR-FAB: Calcd for [C_44_H_65_Cl_2_N_3_O_7_RuSi]^+^: 947.3012; Found: 947.3004.

### 3.4. Preparation of the organic-inorganic hybrid silica material **M1** derived from **5**

A solution of ammonium fluoride (80 μL of a 1 M solution, 4.44 mmol H_2_O, 0.08 mmol NH_4_F) and distilled and deionized water (490 μL, 27.22 mmol) in anhydrous EtOH (2.8 mL) was added to a solution of **5** (184 mg, 0.194 mmol) and TEOS 98% (1.620 g, 7.62 mmol) in anhydrous EtOH (5.2 mL). The mixture was manually shaken for a minute to get a homogeneous solution and was left at room temperature without stirring. During the night gelification occurred and the gel was aged for 5 days. It was then powdered and washed successively several times with ethanol and then with dichloromethane. The solid was dried under vacuum (1 mmHg, 60 ºC, overnight), yielding **M1** as a dark green powder (603 mg). ^29^Si-NMR (79.5 MHz, CP-MAS) δ (ppm): -57 (T^2^), -64.1 (T^3^), -92.9 (Q^2^), -102.6 (Q^3^), -111.7 (Q^4^). EA Calcd. for C_38_H_50_Cl_2_N_3_O_4_RuSiO_1.5_·40SiO_2 _(considering complete condensation): 14.09% C, 1.56% H, 1.30% N, 2.19% Cl, 3.12% Ru, 35.54% Si. Found: 9.60% C, 2.55% H, 0.75% N, 1.00% Cl, 31.19% Si. ICP: 0.86%Ru (0.085 mmol Ru/g material). S_BET_: 332 m²/g; pore diameter: 103–111 Å; pore volume: 0.93–0.89 cm³/g.

### 3.5. Ring-closing metathesis reaction on diethyl 2,2-diallylmalonate (6) with hybrid silica material M1

#### 3.5.1. Under conventional conditions. Typical procedure (Table 1, entry 1)

A solution of **6** (25 mg, 0.104 mmol) in anhydrous and degassed dichloromethane (2.1 mL) was added under nitrogen to **M1** (43 mg, 0.085 mol Ru/g, 0.0037 mmol Ru) placed in a Schlenk tube and the mixture was stirred under inert atmosphere at room temperature for 20 h (GC monitoring). The mixture was filtered under nitrogen atmosphere with a cannula and the solid was washed several times with 2 mL portions of anhydrous dichloromethane. The combined filtrates were evaporated to give pure **9** (19.3 mg, 100% conversion by ^1^H-NMR), whose spectroscopic data were coincident with that reported in the literature [43]. ^1^H-NMR (CDCl_3_, 360 MHz) δ (ppm): 5.60 (m, 2H), 4.19 (q, *J* = 7.2 Hz, 4H), 3.00 (m, 4H), 1.24 (t, *J* = 7.2 Hz, 6H). 

#### 3.5.2. Under microwave irradiation. Typical procedure (Table 1, entry 3)

A solution of **6** (25 mg, 0.104 mmol) in anhydrous and degassed dichloromethane (2.1 mL) was added under nitrogen to **M1** (43 mg, 0.085 mol Ru/g, 0.0037 mmol Ru) placed in a microwave vessel which was sealed, placed in the microwave cavity and irradiated to 60 ºC (*PowerMax *option enabled) for 20 min (GC monitoring). The mixture was filtered under nitrogen atmosphere with a cannula and the solid was washed several times with 2 mL portions of anhydrous dichloromethane. The combined filtrates were evaporated to give pure **9** (23.4 mg, 100% conversion by ^1^H-NMR). The catalyst **M1** was dried and reused in a next cycle. Compounds **10** [34] and **11** [34] were obtained from **7** and **8**, respectively, by analogous procedures under the conditions described in Table 1. 

## 4. Conclusions

We have synthesized and characterized a monosilylated Grubbs-Hoveyda ruthenium alkylidene complex, as well as the corresponding material **M1** made by sol-gel cogelification with tetraethoxysilane (TEOS). This material has been assayed as a recyclable catalyst in the ring-closing metathesis reactions of some selected dienes and enynes. Good results have been obtained for the first cycle in dichloromethane at room temperature for dienes **6** and **7 **and under refluxing conditions for enyne **8**. Notably, considerable improvement has been achieved under microwave irradiation conditions. Thus, drastic reduction of the reaction time favoured the recyclability of the material. Under these conditions up to five cycles have been performed on diene **7** to afford **10 **in a very fast and clean reaction. Supported catalyst **M1** is, to our knowledge, the first case described in the literature of a heterogeneous metathesis catalyst prepared by sol-gel co-gelification from a silylated ruthenium alkylidene. 
